# Optimization of TGF-β1-transduced chondrocytes for cartilage regeneration in a 3D printed knee joint model

**DOI:** 10.1371/journal.pone.0217601

**Published:** 2019-05-23

**Authors:** Jiyong Ahn, Seon Ae Kim, Ki Won Kim, Joon Hyuck Oh, Seok Jung Kim

**Affiliations:** Department of Orthopaedic Surgery, College of Medicine, The Catholic University of Korea, Uijenong bu si, Gyeonggi-do, South Korea; Sungkyunkwan University, REPUBLIC OF KOREA

## Abstract

A cell therapy product of transforming growth factor (TGF)-β1-transduced chondrocytes has been commercialized to treat osteoarthritis of the knee via intra-articular injection. The need for arthroscopic application of the cells to simultaneously treat intra-articular pathologies of knee osteoarthritis is increasingly urgent. The purpose of this study was to optimize TGF-β1-transduced chondrocytes for arthroscopic application. The optimal composition of chondrocytes and thrombin was initially determined by measuring the consolidation time of a diverse ratio of chondrocytes and thrombin mixed with 1 ml of fibrinogen. The consolidation time of the diverse ratio of fibrinogen and atelocollagen mixed with the determined optimal ratio of chondrocytes and thrombin was evaluated. The mixture of the determined optimal ratio of TGF-β1-transduced chondrocytes, atelocollagen, fibrinogen, and thrombin was applied to the cartilage defect of the 3D printed knee joint model arthroscopically. The status of the mixture in the defect was then evaluated. Chondrogenic activities of TGF-β1-transduced chondrocytes mixed with atelocollagen were evaluated. The determined ratio of TGF-β1-transduced chondrocytes to thrombin was 8:2 and that of fibrin to atelocollagen was also 8:2. Excellent maintenance of conformation of the mixture of TGF-β1-transduced chondrocytes, atelocollagen, fibrinogen, and thrombin in the cartilage defect of the 3D printed knee joint model was observed arthroscopically. Increased chondrogenic activities were observed in the group of TGF-β1-transduced chondrocytes mixed with atelocollagen. TGF-β1-transduced chondrocytes can be applied arthroscopically to treat cartilage defects of the knee at an optimized mixing ratio of atelocollagen, fibrinogen, and thrombin.

## Introduction

Articular cartilage is a hyaline cartilage that does not possess blood vessels, nerves, or lymphatics.[1] Damage or degeneration of articular cartilage can contribute to the development and progression of osteoarthritis.[2] Treatments for damaged articular cartilage includes microfracture, osteochondral autograft transplantation, and autologous chondrocyte implantation. Among these treatment options, autologous chondrocyte implantation has been considered the most effective.[3–6] However, such treatments focus on focal cartilage defects and are not effective for arthritis treatment.[7,8] Moreover, autologous chondrocyte implantation requires two successive operations, including tissue biopsy and implantation after cell culture.[9] Therefore, autologous chondrocyte implantation is a burdensome treatment for both doctors and patients due to its numerous potential problems.[10]

Recently, stem cell treatments have emerged as a novel alternative to treat focal articular cartilage defects and osteoarthritis.[11,12] Stem cells are mainly derived from the bone marrow or adipose tissue rather than cell culture, and can be obtained from one-step cell concentration by centrifugation.[13,14] For cells obtained from cultures to be used for clinical application, legal and ethical issues need to be resolved, and such cells can currently only be used for research purposes. Cultured cells may also be utilized within clinical trials.

Cells from one-step cell concentration can be injected intra-articularly for osteoarthritis treatment, and good clinical results have been reported in many published studies.[12,15] However, simple intra-articular injection of cells for osteoarthritis of the knee cannot impart treatment benefits because osteoarthritis is associated with intra-articular pathologies, including meniscus tear and degeneration, mechanical osteophyte formation, and deformities.[16]

Cell therapy products must be delivered by arthroscopy to influence the associated intra-articular pathologies and enhance patient recovery.[17] In this regard, using commercialized cell therapy products with proven safety and developing an arthroscopic surgical technique are important for the treatment of focal cartilage defects or arthritis.

Autologous collagen-induced chondrogenesis (ACIC) is a representative single-stage surgical procedure that uses atelocollagen mixed with fibrin gel as a scaffold under arthroscopic CO_2_ gas insufflation.[18] Since the procedure is performed arthroscopically, fast recovery and good results can be expected.[19,20] Recently, a cell therapy product of transforming growth factor (TGF)-β1-expressing chondrocytes has been commercially available for the treatment of knee osteoarthritis. This product is used to reduce inflammation and pain in knee joints arising from osteoarthritis after intra-articular injection.[15]

Therefore, development of arthroscopic application surgical technique is crucial to improve outcomes of these cellular treatments. The purpose of this study was to determine the optimal composition of TGF-β1-transduced chondrocyte-atelocollagen mixture for arthroscopic cartilage repair.

## Materials and methods

### Cell product preparation

A cell therapy product (Kolon Life Science, Gyeonggi-do, Korea) that contains human chondrocytes transduced with TGF-β1 (5.4 × 10^6^/0.5 ml) and healthy non-genetically manipulated human chondrocytes (1.69 × 10^7^/2.5 ml) at a ratio of 1:3 was used. This product is approved by the Korean Food and Drug Administration for the treatment of osteoarthritis. The cells were stored in separate vials at -135°C using CS-10 (BioLife Solutions, Bothell, WA) as a stabilizing medium. Prior to use, the vials were thawed at 37°C and syringes were used to extract the contents.

### Determination of optimal mixing ratio of TGF-β1-transduced chondrocytes, atelocollagen, and fibrin

#### Determination of the thrombin and TGF-β1-transduced chondrocytes mixing ratio

Each injection procedure comprised two syringes, and a total of five sets of injections were prepared. Briefly, 5 1-ml syringes were filled with varying amounts of thrombin (0.5, 0.4, 0.3, 0.2, and 0.1 ml), and the remaining volume was mixed with varying amounts of TGF-β1-transduced chondrocytes (0.5, 0.6, 0.7, 0.8, and 0.9 ml). Each of the 5 syringes was connected to 5 1-ml syringes filled with fibrin (Green Cross, Gyeonggi-do, Korea) through a Y catheter, and the tip was connected with a 19-gauge needle. After dripping 3 drops of each set of injections into the bottom of the flask, consolidation of the mixture in each group was compared after 30 s.

#### Determination of the fibrin and atelocollagen mixing ratio

Each injection procedure comprised two syringes, and a total of four sets of injections were prepared. Briefly, 4 1-ml syringes with varying amounts of fibrin (0.6, 0.7, and 0.8 ml) were prepared, and the remaining volume was mixed with different amounts of atelocollagen (0.4, 0.3, and 0.2 ml) (Ubiosis, Gyeonggi-do, Korea). The three syringes were each prepared to mix with a 1-ml syringe of thrombin-TGF-β1-transduced chondrocyte mixture, and the optimum ratio was determined in a similar manner to that presented in the previous subsection. After adding 3 drops to the bottom of the flask, consolidation of the mixture in each group was compared after 30 s.

### Chondrogenic activity of the mixture of TGF-β1-transduced chondrocytes, thrombin, atelocollagen, and fibrin

#### Bead formation in the mixture of TGF-β1-transduced chondrocytes, thrombin, atelocollagen, and fibrin

After obtaining an optimal composition of TGF-β1-transduced chondrocytes, thrombin, atelocollagen, and fibrin through the aforementioned procedures, gel formation of the mixture was induced after adding three drops into a sterile Petri dish under room temperature for 20 min. The gel beads were mechanically detached from the plate and transferred into 6-well plates and incubated at 5% CO_2_ at temperatures as low as 37°C after the addition of the culture medium.

#### Chondrogenic differentiation in beads

TGF-β1-transduced chondrocyte-atelocollagen mixture beads were incubated for 21 days in the chondrogenic differentiation medium. Chondrogenic differentiation media comprised Dulbecco’s modified Eagle’s medium-high glucose (DMEM-HG; Gibco, Austria) containing 10^−7^ M dexamethasone, 10 ng/ml TGF-β3, 100 μg/ml sodium pyruvate, 40 μg/ml proline, 25 μM ascorbic acid-2-phosphate, 100 U/ml penicillin, 100 μg/ml streptomycin, and 1% (v/v) ITS plus. All reagents except for DMEM-HG were obtained from Sigma-Aldrich (St. Louis, MO). The media were changed twice weekly for 3 weeks.

#### Evaluation of the expression of anabolic factors

Total RNA was isolated from the TGF-β1-transduced chondrocyte-atelocollagen mixture beads, which were cultured in chondrogenic differentiation media for 3 weeks using the RNeasy mini kit (Qiagen, Valencia, CA). Isolated RNA (1 μg) was translated into cDNA using the cDNA reverse transcription kit (Qiagen). The mRNA expression level of type II collagen (COL2A1), aggrecan, and SOX9 was observed with glyceraldehyde-3-phosphate dehydrogenase serving as a housekeeping gene. The sequences of the primers employed for real-time quantitative polymerase chain reaction (RT-qPCR) are listed in Table 1.

**Table 1:** Primers used for RT-PCR analysis of chondrocyte genes.

Gene (accession NO.)	Primer sequence	Product size (in base pairs)
Human Type II Collagen (COL2A1) (J00116.1)	5’-GTT CAC GTA CAC TGC CCT GA-3’ 5’-TGA CCC TCA AAC TCA TGC CTC-3’	162
Human Aggrecan (BC150624.1)	5’-AGT CAC ACC TGA GCA GCA TC-3’ 5’-TCT GCG TTT GTA GGT GGT GG-3’	482
Human Sox9 (NM000.46)	5’-AGG AAG TCG GTG AAG AAC GG-3’ 5’-AAG TCG ATA GGG GGC TGT CT-3’	275
GAPDH (NM002046)	5’-TTG GTA TCG TGG AAG GAC TCA-3’ 5’-TGT CAT CAT ATT TGG CAG GTTT-3’	126

Type II collagen, aggrecan, and SOX9 were examined using RT-qPCR with GoTaq qPCR Master mix (Promega, Madison, WI). The following thermal protocol was used: 95°C for 3 min, followed by 37 cycles of 15 s at 95°C and 30 s at 60°C, and melting curve analysis was performed at the last cycle. Each sample was processed in triplicate. The average ΔCt value of the triplicates was calculated. Relative expression levels for each primer set were expressed as fold changes using the 2-ΔΔCt method.

#### Measurement of bead size

The size of the gel beads incubated in the differentiation medium for 21 days was measured using a ruler. To measure the bead size, photographs of each bead were taken with a digital camera (Canon, Tokyo, Japan). The results were analyzed statistically using Excel (Microsoft Corporation, Redmond, WA).

#### Histological and immunohistochemical analysis of phenotype differentiation

The TGF-β1-transduced chondrocyte-atelocollagen mixture beads were fixed in 10% formalin (Sigma-Aldrich) after removal of the culture medium at 21 days of culture. Using a graded series of alcohol concentrations (100%, 90%, 80%, 70%, and 60%), the dehydrated fixed beads were embedded in paraffin and made into paraffin blocks. The paraffin blocks were sliced into 5-μm thickness and stained with hematoxylin-eosin, Alcian blue (pH 2.5), and toluidine blue.

Immunohistochemical analysis of type II collagen using the VECTASTAIN ABC Kit (PK-6200; Vector Laboratories, Burlingame, CA) was performed using rabbit polyclonal anti-human collagen type II antibody (ab34712; Abcam, Cambridge, United Kingdom). The 5-μm slices of the tissue were deparaffinized using xylene and alcohol. To block the endogenous peroxidase activity, the tissue slices were incubated with 0.3% H_2_O_2_ in methanol for 30 min. After washing thrice with phosphate-buffered saline for 5 min, the tissue slices were kept in normal blocking serum for 20 min at room temperature. After removal of the normal blocking serum, the tissue slices were incubated at 20°C with type II collagen antibody (diluted to 1:200) in a humidified chamber for 2 h. After removal of the primary antibody and washing with phosphate-buffered saline thrice for 5 min, the slices were incubated with a biotinylated secondary antibody and streptavidin-peroxidase, and peroxidase activity was detected using the Vector DAB substrate kit (3,3-diaminobenzidine) (SK-4100; Vector Laboratories). The stained tissue slices were dehydrated using alcohol and xylene and mounted for microscopic evaluation.

#### Microstructures in the TGF-β1-transduced chondrocyte-atelocollagen mixture beads

The microarchitecture of the TGF-β1-transduced chondrocyte-atelocollagen mixture beads cultured in chondrogenic medium was investigated using scanning electron microscopy and transmission electron microscopy. Briefly, the beads were fixed in Karnovsky fixative (2% glutaraldehyde, 2% paraformaldehyde; Sigma-Aldrich) overnight and then washed twice for 30 min with 0.1 M phosphate buffer.

For scanning electron microscopy, the fixed sample was washed with 0.1 M phosphate buffer for 10 min and dehydrated from using a low to high density alcohol gradient (50%, 60%, 70%, 80%, 90%, 95%, and 100%). After transition using isopentyl acetate, critical point drying (EM CPD300; Leica Microsystems, Austria) was performed for 30 min to 1 h. The sample was observed using field emission scanning electron microscopy (Merlin; Carl Zeiss AG, Oberkochen, Germany) after coating with an ion coater (EM ACE600; Leica Microsystems).

For examination using transmission electron microscopy, the fixed sample was washed with 0.1 M phosphate buffer for 10 min and dehydrated in a graded series of alcohol from low to high density (50%, 60%, 70%, 80%, 90%, 95%, and 100%). The sample was incubated overnight at 4°C in a series of graded EPON (EPON 812, MNA, DDSA, DMP30) and propylene oxide solutions at ratios of 1:4, 1:1, and 3:1 each. The sample was incubated again overnight at 4°C in 100% EPON.

The blocks were embedded in EPON, and 200–250-nm thin sections were cut with an ultramicrotome. The slices were stained using 1% toluidine blue and re-trimmed for electron microscopic observation. Ultra-thin-sectioned slices were placed on the copper grid, stained with uranyl acetate (6%) and lead citrate, and examined using a transmission electron microscope (JEM-1011; JEOL, Tokyo, Japan) at 80 KV.

#### Cell distribution and viability in the TGF-β1-transduced chondrocyte-atelocollagen mixture beads

Viable and dead cells were distinguished in the slices of TGF-β1-transduced chondrocyte-atelocollagen mixture beads using fluorescent dyes. The gel beads were sliced into 1-mm thick sections to which 2 μM calcein acetoxymethyl ester and 4 μM ethidium homodimer (Sigma-Aldrich) were added. The beads were observed under a fluorescence microscope after 30 min.

### Production of the joint model

Data from the magnetic resonance images of the knee’s articular cartilage defect were analyzed and the articular cartilage, femur, patella, tibia, and skin were identified. Image segmentation of the part to be made into a 3D model was performed based on the data from T2 magnetic resonance imaging of a patient with a cartilage defect. Segmented images were converted to stereolithography format, which can be employed for 3D printing.

Printing of the joint model was performed using an OBJECT 260 CONNEX3 3D printer (Stratasys Direct Manufacturing, Los Angeles, CA). The configuration was made after simultaneous emission of photopolymer resin and supporter material in gel form. Resin hardening was completed by ultraviolet application to express suitable fine features and the excellent surface of the joint model (Coraline Soft, Seoul, Korea).

#### Statistical analysis

Statistical analysis was performed using GraphPad prism software (version 5.01, GraphPad Software, San Diego, CA). Comparisons between the three groups were performed with 2-way analysis of variance and t-tests. P values <0.05 were considered to be significant. Data are presented as means ± standard deviation.

## Results

### TGF-β1-transduced chondrocyte preparation

Human chondrocytes transduced with TGF-β1 (5.4 × 10^6^/0.5 ml) were mixed with healthy non-genetically manipulated human chondrocytes (1.69 × 10^7^/2.5 ml). The mixed vial was centrifuged at 500 g for 3 min, and 1.4 ml of the supernatant was aspirated and discarded. The remaining cells and media were used for the experiments. The number of cells employed for the experiment was 5.95 × 10^6^/0.8 ml.

### Optimal ratio of mixture

#### Determination of the thrombin and TGF-β1-transduced chondrocyte mixing ratio

The beads’ consolidation rate, conformation, and texture were observed by employing different ratios of TGF-β1-transduced chondrocytes and thrombin 1 ml of fibrin. The beads’ shape was not maintained, and the consolidation rate was extremely slow at a ratio of 0.9 ml of TGF-β1-transduced chondrocytes to 0.1 ml of thrombin. The mixture did not maintain the bead shape and flowed down after 30 s in the flask.

**Fig 1:**
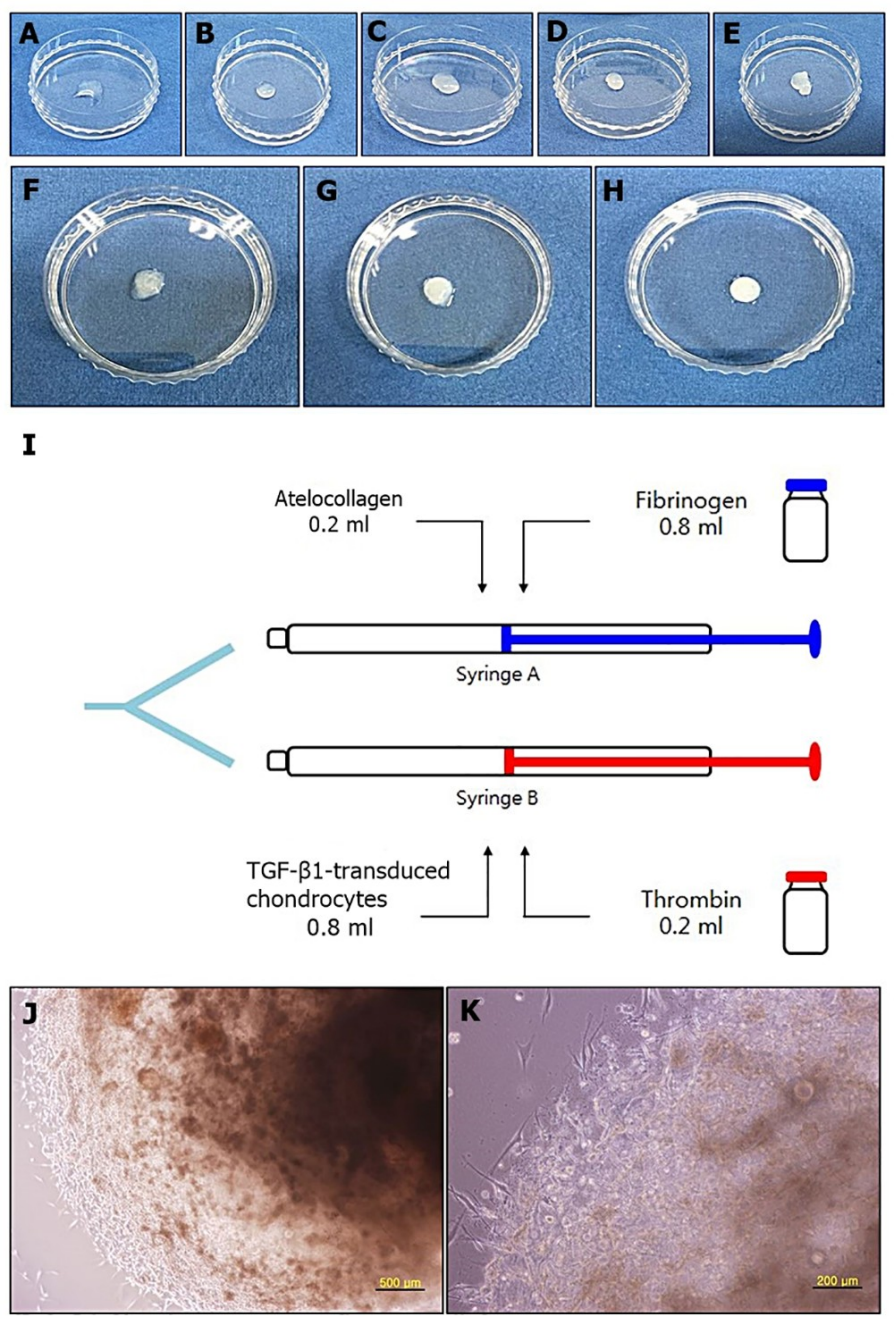
Determination of the thrombin and TGF-β1-transduced chondrocytes mixing ratio. (A) The mixture comprising 0.9 ml of TGF-β1-transduced chondrocytes and 0.1 ml of thrombin did not retain the shape of the beads and flowed down. (B) The mixture comprising 0.8 ml of TGF-β1-transduced chondrocytes and 0.2 ml of thrombin maintained the bead shape well and became firm at approximately 30 s. Mixtures comprising different ratios of TGF-β1-transduced chondrocytes and thrombin: (C) 0.7 ml of TGF-β1-transduced chondrocytes and 0.3 ml of thrombin, (D) 0.6 ml of TGF-β1-transduced chondrocytes and 0.4 ml of thrombin, and (E) 0.5 ml of TGF-β1-transduced chondrocytes and 0.5 ml of thrombin exhibited rapid bead formation. Manipulating these beads was difficult. Determination of the fibrin and atelocollagen mixing ratio: (F) mixing ratios of 0.6 ml of fibrin and 0.4 ml of atelocollagen, (G) 0.7 ml of fibrin and 0.3 ml of atelocollagen (failed to maintain the bead shape at approximately 30 s), and (H) 0.8 ml of fibrin and 0.2 ml of atelocollagen (maintained the bead shape well at around 30 s). Determination of the mixing ratio of TGF-β1-transduced chondrocytes, thrombin, atelocollagen, and fibrin mixture: (I) Two syringes were prepared as one set of injection, and four sets of injection were prepared. Microscopic findings of TGF-β1-transduced chondrocyte-atelocollagen mixture: TGF-β1-transduced chondrocytes were well distributed in the atelocollagen mixture beads, and the round shape of the beads was well maintained after 21 days of culture. Cells exhibited proliferation activity around the mixture beads. (J) × 40 (K) × 100.

Consolidation started immediately after the mixture was placed in the flask at ratios of 0.5 ml of TGF-β1-transduced chondrocytes to 0.5 ml of thrombin, and 0.6 ml of TGF-β1-transduced chondrocytes to 0.4 ml of thrombin. Manipulating the beads at a ratio of 0.7 ml of TGF-β1-transduced chondrocytes to 0.3 ml of thrombin was difficult. The conformation of the bead was maintained well, and hardening was complete in appropriate time at a ratio of 0.8 ml of TGF-β1-transduced chondrocytes to 0.2 ml of thrombin (Fig 1A, 1B, 1C, 1D and 1E).

#### Determination of the fibrin and atelocollagen mixing ratio

The beads’ consolidation rate and maintenance of conformation were observed by employing different ratios of fibrin and atelocollagen holding a ratio of 0.8 ml of TGF-β1-transduced chondrocytes to 0.2 ml of thrombin.

Consolidation time was extremely long, and the conformation of the beads was not maintained at ratios of 0.6 ml of fibrin to 0.4 ml of atelocollagen, and 0.7 ml of fibrin to 0.3 ml of atelocollagen. Hardness and maintenance of conformation were best at a ratio of 0.8 ml of fibrin to 0.2 ml of atelocollagen. The shape of the mixture’s beads was well maintained, and the mixture did not flow down after 30 s in the flask. This ratio was determined to be appropriate for arthroscopic procedures (Fig 1F, 1G and 1A).

### Chondrogenic activity of the mixture

#### Bead formation of TGF-β1-transduced chondrocyte-atelocollagen mixture

Three drops of the prepared mixtures (0.8 ml of fibrinogen to 0.2 ml of atelocollagen and 0.8 ml of TGF-β1-transduced chondrocytes to 0.2 ml of thrombin) were placed into a sterile Petri dish and made into TGF-β1-transduced chondrocyte-atelocollagen mixture beads (Fig 1I).

The TGF-β1-transduced chondrocyte-atelocollagen mixture beads were stored at room temperature for about 20 min for gelation. The culture medium was added to cover the gelling beads. On the following day, the gelling beads were carefully detached using a scraper and moved into a 6-well plate. They were incubated in 5% CO_2_ at 37°C. During this time, the beads maintained their round shape and showed active cell proliferation (Fig 1J and 1K).

#### Chondrogenic differentiation in beads

**Fig 2:**
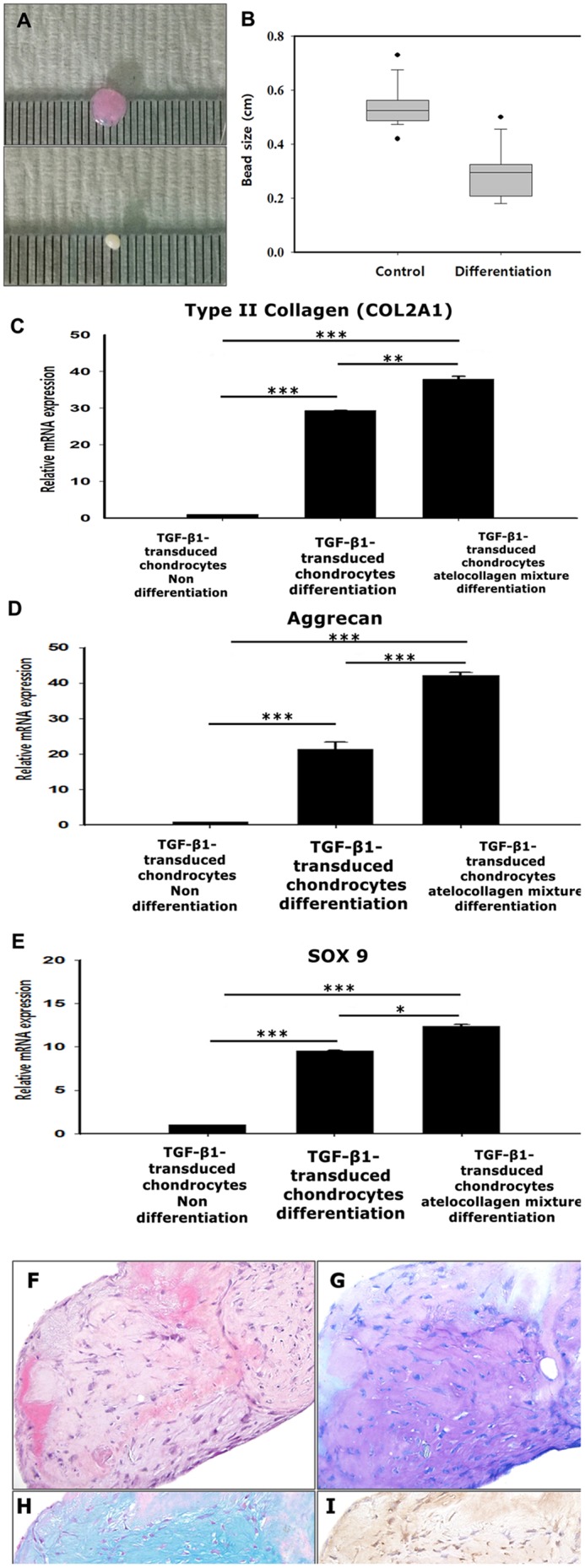
Measurement of bead size. (A) After 21 days of culture, the size of the TGF-β1-transduced chondrocyte-atelocollagen mixture beads (upper) under non-differentiation and (lower) and chondrogenic differentiation conditions was compared. (B) The size of the TGF-β1-transduced chondrocyte-atelocollagen mixture beads under chondrogenic differentiation conditions was significantly reduced. Analysis of gene expression. Real-time quantitative polymerase chain reaction analysis of (C) type II collagen, (D) aggrecan, and (E) SOX9 expression of TGF-β1-transduced chondrocytes cultured for 21 days under non-differentiation and chondrogenic differentiation conditions, and TGF-β1-transduced chondrocytes-atelocollagen cultured for 21 days under chondrogenic differentiation conditions (* P <0.05, ** P <0.01, *** P <0.001). Histological images of the TGF-β1-transduced chondrocyte-atelocollagen mixture: (F) Hematoxylin-eosin, (G) toluidine blue, (H) Alcian blue pH 2.5, and (I) type II collagen. (F-I, × 400).

TGF-β1-transduced chondrocyte-atelocollagen mixture beads were divided into two groups on the following day. One group of beads was processed using DMEM without chondrogenic differentiation medium, while the other was processed with chondrogenic differentiation medium. During the 21 days of culture, no changes in the size of the beads were observed within the initial 7 days. Later, the beads in the chondrogenic differentiation medium became smaller, and the difference between the groups was statistically significant. The beads were also attached to each other, forming a lump that became harder with time (Fig 2A and 2B).

#### Evaluation of anabolic factor expression

We compared the chondrogenic activities between TGF-β1-transduced chondrocytes and the TGF-β1-transduced chondrocyte-atelocollagen mixture. The TGF-β1-transduced chondrocyte bead was a mixture of 1.0 ml of fibrinogen and 0.8 ml of TGF-β1-transduced chondrocytes to 0.2 ml of thrombin. The TGF-β1-transduced chondrocytes-atelocollagen bead was made from a mixture of 0.8 ml of fibrinogen to 0.2 ml of atelocollagen, and 0.8 ml of TGF-β1-transduced chondrocytes to 0.2 ml of thrombin.

RT-qPCR of the TGF-β1-transduced chondrocyte beads and TGF-β1-transduced chondrocytes-atelocollagen beads was performed after 21 days of culture under chondrogenic differentiation conditions. The RT-qPCR revealed upregulation of chondrogenesis-specific markers such as type II collagen (COL2A1), SOX9, and aggrecan (ACAN in TGF-β1-transduced chondrocytes-atelocollagen beads compared with TGF-β1-transduced chondrocytes beads). A much higher mRNA level of type II collagen (COL2A1), SOX9, and aggrecan (ACAN) was observed in TGF-β1-transduced chondrocytes-atelocollagen beads. A significant increase in the mRNA level of type II collagen and aggrecan was found in the TGF-β1-transduced chondrocytes-atelocollagen beads (Fig 2C and 2E).

#### Histology and immunohistochemistry analysis of phenotype differentiation

The mixture of TGF-β1-transduced chondrocytes and atelocollagen beads was cultured under chondrogenic culture conditions. Subsequently, the beads were stained with Alcian blue (pH 2.5), toluidine blue, and hematoxylin-eosin. Immunohistochemical staining for type II collagen was performed at 21 days of culture.

Histological sections showed a chondrocyte and lacuna morphology under chondrogenic culture conditions. Accumulation of cartilage like-extracellular matrix and sulfated glycosaminoglycan was observed at a high rate. Type II collagen expression was also confirmed (Fig 2F, 2G, 2H and 2I).

#### Cell distribution and viability in TGF-β1-transduced chondrocyte-atelocollagen mixture beads

**Fig 3:**
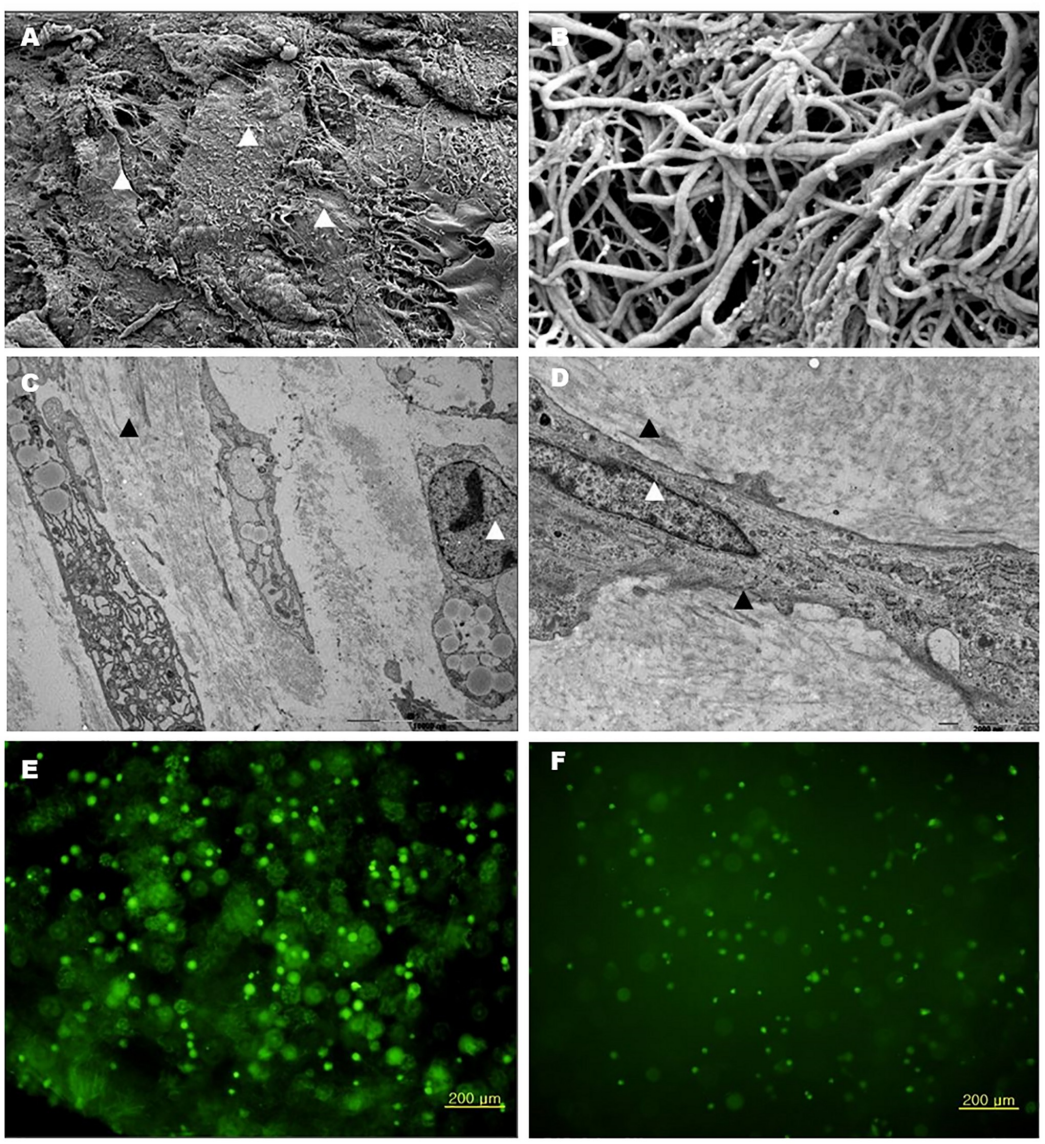
Scanning electron microscopy of TGF-β1-transduced chondrocyte-atelocollagen mixture. (A), (B) TGF-β1-transduced chondrocyte-atelocollagen mixture after 21 days of culture. A large number of cells surrounded the mixture bead and were in close contact with each other (white arrowhead). Profound fibers with striation, which is specific for collagen fiber, and formation of mesh structure for cell attachment were observed. Transmission electron microscopy of TGF-β1-transduced chondrocyte-atelocollagen mixture: (C), (D) TGF-β1-transduced chondrocyte-atelocollagen mixture after 21 days of culture. Numerous vesicles (white arrowhead) in the cytoplasm indicated the active secretory function of cells and production of proteoglycan granules and fibrillar collagens with striation (black arrowhead) for matrix formation. Biochemical staining of TGF-β1-transduced chondrocyte-atelocollagen mixture: viable cells appeared green and dead cells appeared red after staining with calcein acetoxymethyl ester and ethidium homodimer. (E) At 48 h and (F) 7 days after fluorescent dye staining. Original magnification × 100.

The viability of cells was identified from sliced TGF-β1-transduced chondrocyte-atelocollagen mixture beads using calcein acetoxymethyl ester and ethidium homodimer. To observe the viability of cells through fluorescence microscopy, gel beads were sliced at 1-mm thickness. Viable cells exhibited a green tinge and were evenly distributed (Fig 3E). For 7 consecutive days of observation, well-maintained cell survival and conformation of beads were observed although the fluorescence diminished (Fig 3F).

#### Microstructures in TGF-β1-transduced chondrocyte-atelocollagen mixture beads

Scanning electron microscopy showed that the cells in the bead were evenly distributed, and numerous cells surrounded the bead. Collagen fibers with many striations forming spaces and structures for cell attachment were noted (Fig 3A and 3B). Transmission electron microscopy revealed that the surrounding cells formed a layer around each other and actively produced collagen and matrix vesicles (Fig 3C and 3D).

### Simulated arthroscopic injection of TGF-β1-transduced chondrocyte-atelocollagen mixture into the cartilage defect of the joint model

**Fig 4:**
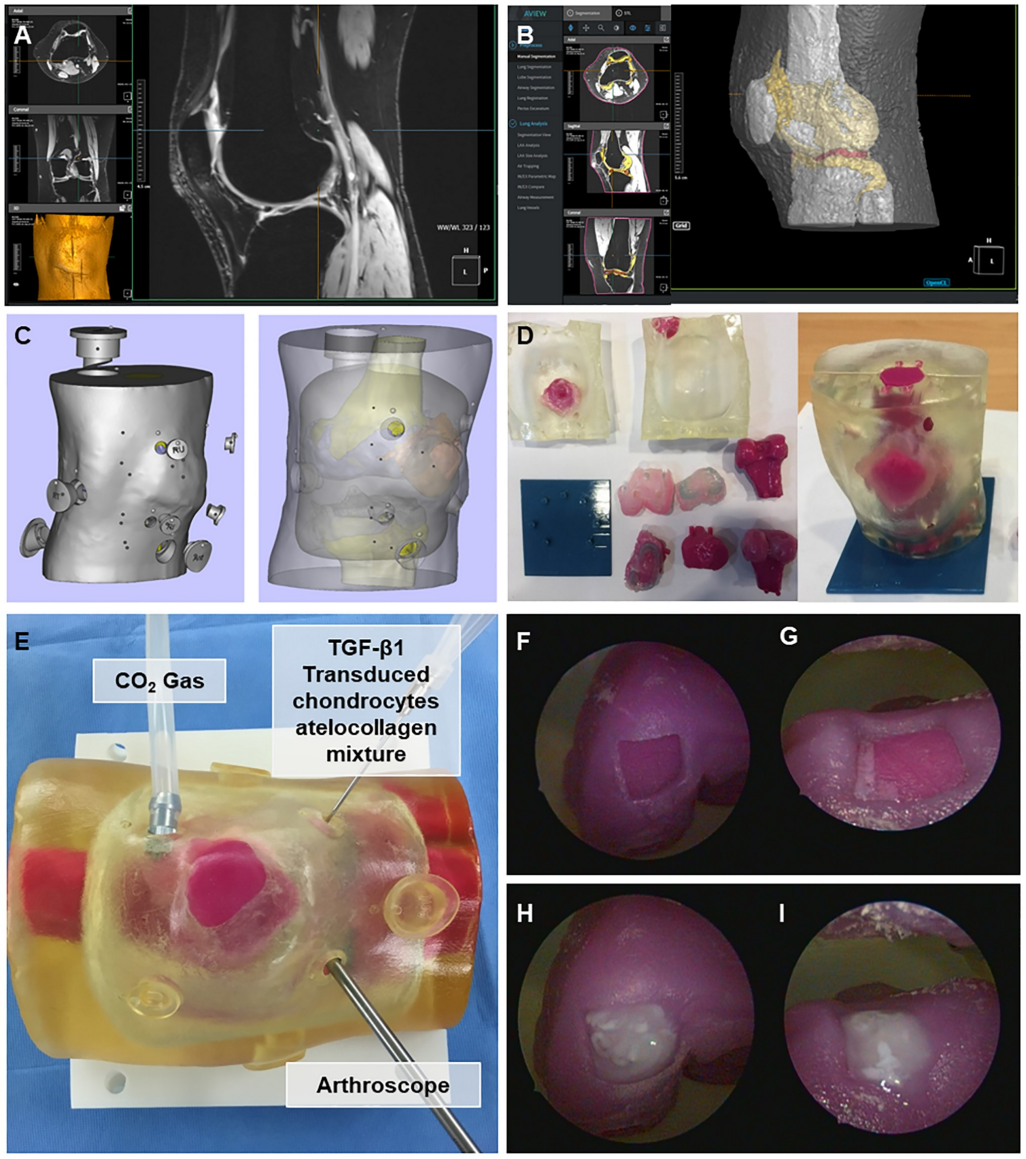
Process of knee joint model production using 3D printing technique. (A) Image segmentation based on the knee T2 magnetic resonance imaging data, (B) conversion of each divided part to stereolithography files, (C) implementation of functional elements with computer-aided design, and (D) output using a 3D printer. Arthroscopic application of TGF-β1-transduced chondrocyte-atelocollagen mixture. (E) Knee joint model from the 3D printing technique. Arthroscopic findings after applying the TGF-β1-transduced chondrocyte-atelocollagen mixture: (F) Medial femoral condyle defect, (G) trochlear defect, (H) application of the mixture to the medial femoral condyle defect, and (I) application to the trochlear defect.

The MRI data were converted to stereolithography files that can be used for 3D printing (Fig 4A and 4B). Functional components, such as the arthroscopic portal, knee joint cavity, and cartilage defect were produced using computer-aided design (CAD) (Fig 4C and 4D). Articular cartilage defects to simulate the injection of TGF-β1-transduced chondrocyte-atelocollagen mixture and a space between the femur, tibia, and patella were created for arthroscope insertion (Fig 4E). To reduce gas leakage, a portal cap was made, and the outer surface was surrounded by an elastic material to mimic skin texture.

The 3D printed knee joint model was installed on the operating table and the portable arthroscope and instruments were prepared for the procedure. The CO_2_ gas insufflation instrument was placed in the superolateral portal, and the cartilage defect was examined through the anterolateral or anteromedial portal (Fig 4E). Carbon dioxide (CO_2_) was insufflated at a pressure of 20 mmHg and flow rate of 20 l/min using a Wolf cannula (Karl Storz GmbH, Tuttlingen, Germany) and disposable tubing with a filter (Insufflation tubing with Wolf adaptor; Leonhard Lang UK Ltd., Stroud, United Kingdom) through a superolateral portal.

The maintenance of bead conformation after injection of the mixture into the cartilage defect was observed by arthroscopy. After 3 min, stable attachment of the injected mixture to the defect was confirmed by arthroscopy after rotating the knee joint model in full 360° turns several times (Fig 4F, 4G, 4H and 4I).

## Discussion

This study was performed to demonstrate the advantages of TGF-β1-transduced chondrocytes, atelocollagen, fibrin, and thrombin injections for maximizing the efficacy of the cartilage regeneration procedure. The optimized ratio of TGF-β1-transduced chondrocytes, atelocollagen, fibrin, and thrombin was determined by carrying out several theoretically possible combination experiments using the materials used for arthroscopic surgery.

Surgical articular cartilage repair is based on arthroscopic microfracturing.[21] The microfracture method is advantageous because it requires a simple arthroscopic technique, is easy to perform, and carries a low cost, even when quality and quantity of regenerative tissues are insufficient.[22]

Considerable efforts have been exerted to use various materials such as collagen, hyaluronic acid, and chitosan, as a scaffold to maximize the effects of the arthroscopic microfracture technique.[23,24] One of the most widely used scaffolds is collagen.[25] A relatively long-term study on autologous matrix-induced chondrogenesis showed the superiority of collagen as a scaffold for regeneration of joint cartilage.[26]

Among the arthroscopic surgical repair techniques for articular cartilage defects, ACIC is an innovative method to treat joint cartilage lesion without an open incision. In fact, all operative steps, from the preparation of the lesion to implantation of the scaffold, are performed using arthroscopy.[27]

A combined product of normal cartilage cells and transduced cells overexpressing TGF-β1 has been approved for the treatment of Kellgren-Lawrence grade 3 knee osteoarthritis via intra-articular injection. The specific target of this treatment is early and moderately advanced osteoarthritis. TGF-β1 has been speculated to stimulate chondrogenesis and the growth of articular chondrocytes.[28] It has significant roles in tissue regeneration, cell differentiation, and extracellular matrix protein synthesis.[29] Preclinical studies and placebo-controlled trials in patients with arthritis of the knee showed promising results of TGF-β1-transduced chondrocytes.[15]

Synovial macrophages are located in the synovial lining and release inflammatory mediators that in turn induce the release of matrix metalloproteinases (MMPs) and aggrecanases, among others, from synovial fibroblasts, leading to cartilage damage. [30] In the cartilage tissue, TGF-β1 is secreted in an inactive form and stored in the extracellular matrix where MMP-3 may stimulate its activation. However, TGF-β1-transduced chondrocytes do not act through this mechanism and the secreted TGF-β1 directly stimulates proliferation of a diverse group of cells within the joint. [31]

This localized delivery of TGF-β1 and normal chondrocytes stimulates interleukin (IL)-10 production and M2 macrophage polarization, which creates an anti-inflammatory environment and leads to chondrogenesis in the knee joint.[32]. TGF-β itself stimulates cartilage repair through stimulation of extracellular matrix synthesis and chondrogenesis in the synovial lining, chondrocytes and surrounding stem cells, while decreasing the catabolic activity of IL-1.[31]

However, injection of cells into the joint would not realize the maximal treatment effect of TGF-β1-transduced chondrocytes in osteoarthritis. The pathophysiology of osteoarthritis involves cartilage defects, osteophyte formation, torn meniscuses, degenerated ligaments, and synovial hypertrophy.[33] Hence, these pathologic conditions should also be addressed for effective osteoarthritis treatment.[34]

To expand the indications and maximize the effectiveness of this cell therapy product, an arthroscopic procedure that involves excision of osteophytes and inflamed tissues, debridement of denuded articular cartilage with sclerotic subchondral bone and microfracture, and arthroscopic delivery of the cell therapy product is essential.[21]

After the preparation of osteoarthritic joint lesions, injection of TGF-β1-transduced chondrocytes with a scaffold by arthroscopy can be a novel treatment option for osteoarthritis. The arthroscopic procedure can be performed in a single-stage, similar to ACIC and mesenchymal-cell-induced-chondrogenesis.[27,35] The present study provides data on the proper mixing ratio between manufactured cells and scaffold for single-stage arthroscopic procedure.

Recent conceptions of osteoarthritis development state that wear and tear of the intra-articular structure is not the sole cause, and immune and inflammatory reactions are also contributing factors.[34,36] Inflammation and immune reactions also play a significant role in the progression of the disease.[37] Allogeneic human chondrocytes expressing TGF-β1 could modulate the inflammatory milieu in the knee joint inflammation and provide a suitable environment for damaged cartilage regeneration.[38] Non-transduced allogeneic human chondrocytes are also expected to contribute to effective cartilage regeneration.[38]

Therefore, if a mixture of normal and TGF-β1-expressing chondrocytes is used, the normal chondrocytes could participate in cartilage repair directly while TGF-β1 creates an anti-inflammatory and regeneration-conducive environment through suppression of inflammatory mediators and stimulation of the synthetic activity of chondrocytes, synovial cells, and stem cells in the knee joint.

Atelocollagen and TGF-β1-transduced chondrocytes were mixed and incubated to determine whether atelocollagen would be helpful in cartilage regeneration. Enhancement in the chondrogenic activity of TGF-β1-transduced chondrocytes was observed after mixing with atelocollagen compared to preparations without a scaffold. The number of cells and expression of type 2 collagen and aggrecan also increased. The atelocollagen used in this study showed a good, natural collagen structure when observed with electron microscopy. The collagen structure can be damaged or denatured by enzymatic dissolution of raw material.[39,40] However, in this study, the atelocollagen gel was produced by salt precipitation, which can minimize injury to the collagen fibers and preserve the natural collagen structure.[39,40] Atelocollagen also acts as a cell carrier and enhances chondrogenesis through stimulation of the chondrocyte mixture.

As a clinical simulation, a mixture of these materials at an optimized ratio was injected arthroscopically under CO_2_ insufflation into the cartilage defect of 3D-printed knee joint models. The injected mixture was consolidated in 20–30 s, which gave sufficient time for the surgeon to manipulate the mixture and adjust the conformation.

Had the consolidation time been shorter than 20–30 s, manipulation of the implanted mixture would have been difficult. In contrast, if the consolidation time was longer than 20–30 s, rolling down of the implanted mixture would inhibit effective repair of the cartilage defect. Therefore, an optimized mixing ratio for arthroscopic application was determined to allow for proper placement and achievement of good cartilage. The use of 3D-printed joint models for simulation training on arthroscopic procedures has some advantages, namely, 1) almost perennial reuse of the models, 2) avoidance of ethical issues, and 3) low cost. Thus, these models offer an advantageous alternative to cadaver models for developing surgical procedures.

## Conclusions

Our study provides an appropriate mixing ratio of TGF-β1-transduced chondrocytes, atelocollagen, fibrin, and thrombin. The results also present preliminary data on the surgical application of TGF-β1-transduced chondrocytes based on clinical simulation using 3D-printed joint models. TGF-β1-transduced chondrocytes can be applied arthroscopically to treat cartilage defects of the knee at an optimized mixing ratio of atelocollagen, fibrinogen, and thrombin.
